# Supplementary material for: Infliximab for Treatment of Immune Adverse Events and Its Impact on Tumor Response
Source: Cancers (Basel). 2023 Oct 27;15(21):5181. doi: 10.3390/cancers15215181 (PMC10649345; doi:10.3390/cancers15215181)
Supplement: Supplementary file 1 [file cancers-15-05181-s001.zip › cancers-2642925-supplementary.pdf]

Supplementary Table S1: Presence of each adverse event for all patients in all subgroups of therapy and cancers

AE	All patients (n=185)	Monotherapy ICI (n=76)	Combo ICI (n=109)	Melanoma patients (n=93)		GU cancer patients (n=37)	
				Mono (n=24)	Combo (n=69)	Mono (n=19)	Combo (n=18)
	N (%)	N (%)	N (%)	N (%)	N (%)	N (%)	N (%)
Colitis	121 (65.4%)	42 (55.3%)	79 (72.5%)	18 (75%)	57 (82.6%)	12 (63.2%)	12 (66.7%)
Pneumonitis	29 (15.7%)	15 (19.7%)	14 (12.8%)	1 (4.2%)	4 (5.8%)	1 (5.3%)	2 (11.1%)
AIN	10 (5.4%)	5 (6.6%)	5 (4.6%)	1 (4.2%)	4 (5.8%)	0 (0%)	1 (5.6%)
Myocarditis	9 (4.9%)	6 (7.9%)	3 (2.8%)	1 (4.2%)	1 (1.4%)	4 (21.1%)	2 (11.1%)
Myositis	9 (4.9%)	4 (5.3%)	5 (4.6%)	0 (0%)	1 (1.4%)	3 (15.8%)	1 (5.6%)
Encephalitis	6 (3.2%)	1 (1.3%)	5 (4.6%)	1 (4.2%)	5 (7.2%)	0 (0%)	0 (0%)
Arthritis	2 (1.1%)	1 (1.3%)	1 (0.9%)	0 (0%)	1 (1.4%)	0 (0%)	0 (0%)
Dermatitis	2 (1.1%)	1 (1.3%)	1 (0.9%)	1 (4.2%)	0 (0%)	0 (0%)	0 (0%)
Neuritis	2 (1.1%)	0 (0%)	2 (1.8%)	0 (0%)	2 (2.9%)	0 (0%)	0 (0%)
HLH	1 (0.5%)	1 (1.3%)	0 (0%)	0 (0%)	0 (0%)	0 (0%)	0 (0%)
Necrolysis	1 (0.5%)	0 (0%)	1 (0.9%)	0 (0%)	0 (0%)	0 (0%)	0 (0%)
Neuritis	1 (0.5%)	0 (0%)	1 (0.9%)	0 (0%)	1 (1.4%)	0 (0%)	0 (0%)
Uveitis	1 (0.5%)	1 (1.3%)	0 (0%)	1 (4.2%)	0 (0%)	0 (0%)	0 (0%)
Arthralgia	1 (0.5%)	1 (1.3%)	0 (0%)	1 (4.2%)	0 (0%)	0 (0%)	0 (0%)
Papillitis	1 (0.5%)	1 (1.3%)	0 (0%)	0 (0%)	0 (0%)	1 (5.3%)	0 (0%)
Mucositis	1 (0.5%)	1 (1.3%)	0 (0%)	1 (4.2%)	0 (0%)	0 (0%)	0 (0%)
Neurotoxicity	1 (0.5%)	1 (1.3%)	0 (0%)	1 (4.2%)	0 (0%)	0 (0%)	0 (0%)
Pancreatitis	1 (0.5%)	0 (0%)	1 (0.9%)	0 (0%)	1 (1.4%)	0 (0%)	0 (0%)
Polyarthritis	1 (0.5%)	0 (0%)	1 (0.9%)	0 (0%)	1 (1.4%)	0 (0%)	0 (0%)

AIN, acute interstitial nephritis; CVD, cardiovascular disease; DM, diabetes mellitus; HLD, hyperlipidemia; HTN, hypertension; GU, genitourinary.

Supplementary Table S2: Predictors of response to infliximab within 3 months

Covariate	Level	Univariate logistic model		Multivariate logistic model	
		OR (95% CI)	P-value	OR (95% CI)	P-value
Age at ICI initiation	In 1 unit	0.994 (0.970-1.019)	0.6320		
Baseline creatinine	In 1 unit	1.901 (0.537-6.735)	0.3193		
Time from ICI to irAE	In 1 unit	1.036 (0.976-1.100)	0.2414		
Time from irAE to infliximab	In 1 unit	0.998 (0.991-1.006)	0.6576		
Sex	Female	1.000			
	Male	0.811 (0.382-1.720)	0.5847		
ICI combination/monothera	Monotherapy	1.000		1.000	
	Combo	1.896 (0.981-3.662)	0.0569	1.539 (0.762-3.107)	0.2295
Cancer type	GU	1.000			
	Melanoma	1.049 (0.443-2.485)	0.9127		
	Other	0.856 (0.337-2.177)	0.7448		
Steroids before infliximab	No	1.000			
	Yes	0.692 (0.347-1.382)	0.2972		
HTN	No	1.000			
	Yes	0.639 (0.318-1.284)	0.2082		
DM	No	1.000			
	Yes	0.596 (0.293-1.212)	0.1529		
HLD	No	1.000			
	Yes	0.889 (0.450-1.757)	0.7348		
CVD	No	1.000			
	Yes	0.704 (0.321-1.543)	0.3805		
Hypothyroid	No	1.000			
	Yes	0.978 (0.481-1.992)	0.9521		
Autoimmune disease	No	1.000			
	Yes	1.146 (0.297-4.418)	0.8427		
AKI prior to infliximab	No	1.000			
	Yes	0.972 (0.453-2.086)	0.9416		
AKI within 1 month of infliximab	No	1.000			
	Yes	0.562 (0.284-1.114)	0.0988		
Colitis	No	1.000		1.000	
	Yes	4.887 (2.441-9.786)	<.0001	4.59 (2.275-9.262)	<.0001
AIN	No	1.000			
	Yes	0.453 (0.116-1.759)	0.2523		
Pneumonitis	No	1.000			
	Yes	0.213 (0.092-0.492)	0.0003		
Encephalitis	No	1.000			
	Yes	0.558 (0.090-3.442)	0.5297		
Myocarditis	No	1.000			
	Yes	0.453 (0.116-1.759)	0.2523		
Myositis	No	1.000			
	Yes	0.281 (0.072-1.094)	0.0671		

AKI, acute kidney injury; AIN, acute interstitial nephritis CVD, cardiovascular disease; DM, diabetes mellitus; HLD, hyperlipidemia; HTN, hypertension; ICI, immune checkpoint inhibitor; irAE, immune-related adverse event.

Supplementary Table S3: Cohorts' progression-free survival (PFS) from infliximab initiation until disease progression or death, whichever occurred first; those who were alive without progression (based on response evaluation after infliximab initiation) or death were censored at the time of response evaluation), Melanoma (median PFS=9.7 months; 95% CI 6.7-17.6) and Genitourinary (median PFS=4.4 months; 95% CI 3.2-11.7)

Covariate	Level	Melanoma (Median PFS=9.7 months; 95% CI 6.7-17.6)				Genitourinary (Median PFS=4.4 months; 95% CI 3.2-11.7)			
		Univariate Cox model		Multivariate Cox model		Univariate Cox model		Multivariate Cox model	
		HR (95% CI)	P-value	HR (95% CI)	P-value	HR (95% CI)	P-value	HR (95% CI)	P-value
Age ICI	In 1 unit change	1.006 (0.986-1.027)	0.5281			1.010 (0.972-1.050)	0.6101		
Baseline creatinine	In 1 unit change	0.446 (0.097-2.042)	0.2981			0.459 (0.122-1.725)	0.2490		
Time from ICI to irAE	In 1 unit change	0.982 (0.936-1.030)	0.4540			0.905 (0.790-1.036)	0.1485		
Time from irAE to infliximab	In 1 unit change	0.998 (0.991-1.005)	0.6158			0.975 (0.943-1.009)	0.1436		
Sex	Female	1.000				1.000			
	Male	0.671 (0.372-1.208)	0.1833			0.877 (0.319-2.414)	0.7996		
ICI combination /monotherapy	Monotherapy	1.000		1.000		1.000		1.000	
	Combo	1.404 (0.711-2.773)	0.3289	1.156 (0.574-2.326)	0.6851	1.250 (0.533-2.934)	0.6077	1.370 (0.573-3.278)	0.4791
Steroids before infliximab	No	1.000				1.000			
	Yes	0.522 (0.243-1.121)	0.0957			1.216 (0.494-2.990)	0.6707		
HTN	No	1.000				1.000			
	Yes	1.118 (0.619-2.018)	0.7124			1.234 (0.452-3.370)	0.6811		
DM	No	1.000				1.000			
	Yes	0.852 (0.418-1.738)	0.6602			1.093 (0.471-2.538)	0.8358		
HLD	No	1.000				1.000			
	Yes	0.891 (0.451-1.763)	0.7410			1.615 (0.654-3.988)	0.2984		
CVD	No	1.000		1.000		1.000			
	Yes	3.897 (1.998-7.601)	<.0001	3.776 (1.907-7.475)	0.0001	1.172 (0.427-3.213)	0.7578		
Hypothyroid	No	1.000				1.000			
	Yes	0.524 (0.274-1.001)	0.0503			0.507 (0.150-1.715)	0.2746		
Autoimmune disease	No	1.000				1.000			
	Yes	0.939 (0.335-2.638)	0.9056			0.939 (0.335-2.638)	0.9056		
AKI prior to infliximab	No	1.000				1.000			
	Yes	1.660 (0.814-3.383)	0.1632			0.931 (0.379-2.288)	0.8769		
Colitis	No	1.000				1.000		1.000	
	Yes	1.107 (0.531-2.306)	0.7871			0.539 (0.235-1.239)	0.1458	0.516 (0.222-1.200)	0.1245
AIN	No	1.000							

Covariate	Level	Melanoma (Median PFS=9.7 months; 95% CI 6.7-17.6)				Genitourinary (Median PFS=4.4 months; 95% CI 3.2-11.7)			
		Univariate Cox model		Multivariate Cox model		Univariate Cox model		Multivariate Cox model	
		HR (95% CI)	P-value	HR (95% CI)	P-value	HR (95% CI)	P-value	HR (95% CI)	P-value
	Yes	0.748 (0.223-2.503)	0.6374			-			
Pneumonitis	No	1.000				1.000			
	Yes	1.008 (0.294-3.450)	0.9904			2.987 (0.645-13.831)	0.1617		
Encephalitis	No	1.000				1.000			
	Yes	0.980 (0.302-3.185)	0.9737			0.980 (0.302-3.185)	0.9737		

Supplementary Table S4: Cox regression model for overall survival (OS) from infliximab initiation: melanoma (N=93, 43 deaths) and genitourinary cancers (N=37, 20 deaths)

Covariate	Level	Melanoma				Genitourinary			
		Univariate Cox model		Multivariate Cox model		Univariate Cox model		Multivariate Cox model	
		HR (95% CI)	P-value	HR (95% CI)	P-value	HR (95% CI)	P-value	HR (95% CI)	P-value
Age ICI	In 1 unit change	1.010 (0.987-1.033)	0.4173			1.025 (0.980-1.073)	0.2811		
Baseline creatinine	In 1 unit change	1.015 (0.211-4.874)	0.9856			0.527 (0.112-2.471)	0.4164		
Time from ICI to irAE	In 1 unit change	1.001 (0.955-1.050)	0.9521			0.924 (0.813-1.050)	0.2258		
Time from irAE to infliximab	In 1 unit change	0.995 (0.988-1.003)	0.2276			0.983 (0.954-1.013)	0.2621		
Sex	Female	1.000				1.000			
	Male	0.806 (0.436-1.489)	0.4911			1.091 (0.358-3.323)	0.8776		
ICI combination/ monotherapy	Monotherapy	1.000		1.000		1.000		1.000	
	Combo	1.266 (0.622-2.579)	0.5158	1.225 (0.600-2.501)	0.5767	0.741 (0.292-1.880)	0.5282	0.857 (0.333-2.206)	0.7489
Steroids before infliximab	No	1.000				1.000			
	Yes	0.525 (0.243-1.137)	0.1021			1.904 (0.737-4.918)	0.1834		
HTN	No	1.000				1.000			
	Yes	1.117 (0.605-2.063)	0.7229			1.126 (0.369-3.440)	0.8347		
DM	No	1.000				1.000			
	Yes	0.662 (0.317-1.384)	0.2733			1.254 (0.502-3.131)	0.6279		
HLD	No	1.000				1.000			
	Yes	0.829 (0.417-1.647)	0.5924			1.160 (0.449-3.001)	0.7588		
CVD	No	1.000				1.000			
	Yes	1.989 (0.997-3.969)	0.0511			2.106 (0.734-6.041)	0.1659		
Hypothyroid	No	1.000				1.000			
	Yes	0.589 (0.306-1.133)	0.1127			0.574 (0.166-1.992)	0.3821		
Autoimmune disease	No	1.000				1.000			
	Yes	0.622 (0.149-2.591)	0.5142			0.939 (0.335-2.638)	0.9056		
Aki prior to infliximab	No	1.000		1.000		1.000			
	Yes	2.140 (1.019-4.491)	0.0444	2.113 (1.005-4.441)	0.0485	0.700 (0.248-1.974)	0.5003		
Colitis	No	1.000				1.000			
	Yes	0.651 (0.310-1.365)	0.2559			0.610 (0.237-1.571)	0.3061		
AIN	No	1.000							
	Yes	1.489 (0.458-4.833)	0.5079			-			
Pneumonitis	No	1.000				1.000			
	Yes	1.729 (0.598-4.997)	0.3121			1.087 (0.144-8.205)	0.9357		

Covariate	Level	Melanoma				Genitourinary			
		Univariate Cox model		Multivariate Cox model		Univariate Cox model		Multivariate Cox model	
		HR (95% CI)	P-value	HR (95% CI)	P-value	HR (95% CI)	P-value	HR (95% CI)	P-value
Encephalitis	No	1.000				1.000			
	Yes	1.530 (0.470-4.976)	0.4798			0.980 (0.302-3.185)	0.9737		
Myocarditis	No					1.000			
	Yes					2.859 (0.926-8.825)	0.0677		
Myositis	No					1.000		1.000	
	Yes					10.978 (2.871-41.978)	0.0005	10.638 (2.749-41.162)	0.0006

There were only 2 myocarditis AEs and 1 myositis AE, and these variables are not included in univariate Cox regression models.
